# The Understanding of the Plant Iron Deficiency Responses in Strategy I Plants and the Role of Ethylene in This Process by Omic Approaches

**DOI:** 10.3389/fpls.2017.00040

**Published:** 2017-01-24

**Authors:** Wenfeng Li, Ping Lan

**Affiliations:** ^1^Collaborative Innovation Center of Sustainable Forestry in Southern China of Jiangsu Province, College of Biology and the Environment, Nanjing Forestry UniversityNanjing, China; ^2^State Key Laboratory of Soil and Sustainable Agriculture, Institute of Soil Science, Chinese Academy of SciencesNanjing, China

**Keywords:** iron deficiency, transcriptomics, proteomics, co-expression, ethylene

## Abstract

Iron (Fe) is an essential plant micronutrient but is toxic in excess. Fe deficiency chlorosis is a major constraint for plant growth and causes severe losses of crop yields and quality. Under Fe deficiency conditions, plants have developed sophisticated mechanisms to keep cellular Fe homeostasis via various physiological, morphological, metabolic, and gene expression changes to facilitate the availability of Fe. Ethylene has been found to be involved in the Fe deficiency responses of plants through pharmacological studies or by the use of ethylene mutants. However, how ethylene is involved in the regulations of Fe starvation responses remains not fully understood. Over the past decade, omics approaches, mainly focusing on the RNA and protein levels, have been used extensively to investigate global gene expression changes under Fe-limiting conditions, and thousands of genes have been found to be regulated by Fe status. Similarly, proteome profiles have uncovered several hallmark processes that help plants adapt to Fe shortage. To find out how ethylene participates in the Fe deficiency response and explore putatively novel regulators for further investigation, this review emphasizes the integration of those genes and proteins, derived from omics approaches, regulated both by Fe deficiency, and ethylene into a systemic network by gene co-expression analysis.

## Introduction

Iron (Fe) is an essential element for living organisms including plants. Fe-containing proteins play a variety of vital roles in cellular respiration, intermediary metabolism, oxygen transport, and DNA stability and repair, as well as photosynthesis in plants. In human beings, Fe deficiency causes severe healthy problems, including anemia, which affects billions of people worldwide (McLean et al., 2009). Although Fe is an abundant element in the earth's crust, it is one of the least available elements for plants grown on aerobic soils with neutral to basic pH. Approximately 30% of the land worldwide consists of alkaline soils, leading to a demand in bioavailable Fe for plant fitness. As a consequence, Fe deficiency is a major constraint for crop yield and quality, which eventually affects human health via food-chain, particularly to those people whose diets mainly relying on plant resources (Abadia et al., 2011). To cope with Fe deficiency, plants have developed sophisticated mechanisms to keep cellular Fe homeostasis via various physiological, morphological, metabolic, and gene expression changes to facilitate the availability of Fe (Jeong and Guerinot, 2009; Ivanov et al., 2012; Kobayashi and Nishizawa, 2012).

Two distinct strategies of plant Fe uptake mechanisms have been proposed, i.e., Strategy I and Strategy II (Romheld and Marschner, 1986), although a combined strategy has been mentioned mainly because the Strategy II plant rice can also absorb Fe^2+^ (Ricachenevsky and Sperotto, 2014). Grasses use a chelation strategy (Strategy II) to obtain Fe from soil. This process is largely dependent on the release of PhytoSiderophores (PS) by root, which would form stable Fe-PS chelates. These chelates are taken up by a plasma membrane-localized oligopeptide transporter, YELLOW-STRIP1 (Curie et al., 2001). Dicotyledonous plants including model plant *Arabidopsis thaliana* and non-graminaceous monocots mobilize Fe via reduction strategy (Strategy I). The first step in this strategy is the acidification of the rhizosphere mediated by the H^+^-translocating P-type ATPase AHA2 (Santi and Schmidt, 2009; Ivanov et al., 2012), which leads to an increase of the chelated Fe (III) concentration. Subsequently, the root surface-localized ferric chelate reductase FERRIC-REDUCTION OXIDASE2 (FRO2) (Robinson et al., 1999) reduces Fe (III) to soluble Fe (II), which is then taken up into epidermal cells by the Fe-REGULATED TRANSPORTER1 (IRT1) (Eide et al., 1996).

Expression of both FRO2 and IRT1 genes is regulated by the basic helix-loop-helix (bHLH) transcription factor FER-LIKE Fe DEFICIENCY-INDUCED TRANSCRIPTION FACTOR (FIT) (Colangelo and Guerinot, 2004; Bauer et al., 2007). FIT exerts its regulation by forming heterodimers with bHLH38 and bHLH39 (Yuan et al., 2008). Studies have shown that the Ib sub-group of bHLH proteins bHLH100 and bHLH101 are also involved in Arabidopsis Fe deficiency responses by interacting with FIT (Wang et al., 2013) or via a FIT-independent manner (Sivitz et al., 2012). Moreover, the mediator16 (MED16) is reported to regulate the expression of *FRO2* and *IRT1* by interacting with FIT (Yang et al., 2014; Zhang et al., 2014).

There is increasing evidence showing that phytohormones play vital roles in the Fe deficiency response of plants. For example, it has been reported that the expression of *FRO2* and *IRT1* is positively or negatively affected by several hormones, such as auxin, ethylene, cytokinins, jasmonic acid, and brassinosteroids, and other signaling molecules, including nitric oxide (Hindt and Guerinot, 2012; Kobayashi and Nishizawa, 2012). Among them, ethylene has been extensively explored in the involvement of Fe deficiency response by application of ethylene precursors or inhibitors or ethylene related mutants (Romera and Alcantara, 1994; Schmidt et al., 2000; Schikora and Schmidt, 2001, 2002; Schmidt and Schikora, 2001; Zaid et al., 2003; Lucena et al., 2006; Waters et al., 2007; Garcia et al., 2010, 2011; Wu et al., 2011; Kabir et al., 2012; Garcia et al., 2015; Ye et al., 2015). It has been uncovered (Lingam et al., 2011) that ethylene regulates the expression of Fe acquisition genes via modulation of FIT protein stability through the interaction between FIT and ETHYLENE INSENSITIVE3 (EIN3)/ETHYLENE INSENSITIVE3-LIKE1 (EIL1). Transcription factors EIN3 and EIL1, two major downstream players (Chao et al., 1997), once activated by EIN2 (Alonso et al., 1999), could trigger extensive ethylene responses, including the expression changes of ethylene-responsive genes (Zhang F. et al., 2016). Under Fe deficiency, however, only a small portion of differentially expressed genes is ethylene-responsive, the number being much less than that of ethylene-regulated genes (Garcia et al., 2010). Thus, it remains unclear whether EIN2, a key positive regulator mediating ethylene signaling, is required or not to fascinate the interaction between FIT and EIN3/EIL1 under Fe starvation. On the other hand, whether the interaction of FIT and EIN3/EIL1 could stabilize EIN3/EIL1 is unknown, although this interaction could modulate the FIT stability (Lingam et al., 2011). All these studies suggest that the regulatory mechanism of FIT and the interaction of Fe deficiency and ethylene are far from complete.

Over the past 10 years, omic approaches have been widely used to investigate the genome-wide gene expression changes under Fe deficiency. Hundreds of genes, some of which also being annotated as ethylene-responsive genes, are reported to be regulated by Fe status, and these studies have led to the identification of some novel regulators, such as FIT (Colangelo and Guerinot, 2004), POPEYE (Long et al., 2010), MYB72 and MYB10 (Palmer et al., 2013), and others involved in the Fe response. Although the number of detected proteins regulated by Fe deficiency is much less than that the number of transcripts, some robust biological processes have been uncovered by proteomics and some Fe-responsive proteins are not regulated at transcript level, suggesting that the integration of both proteomics and transcriptomics is better to acquire a comprehensive understanding of how plants respond to Fe stress. A comprehensive understanding of the interaction between ethylene and Fe deficiency can be referenced in a recent review (Lucena et al., 2015). To avoid redundancy, ethylene synthesis and signaling under Fe deficiency will not be repeatedly presented in this review, which are available in the recent research topics. By contrast, in this review we would mainly focus on the genes and proteins, which are regulated by both Fe and ethylene, revealed by omic approaches. Given that studies on the regulatory roles of ethylene in the Fe deficiency response in Strategy II species is less and most of the comprehensive results are based on the Arabidopsis research, in this review most results are based on this model plant.

## Morphological and physiological changes under Fe deficiency with or without ethylene

Over last three decades, scientists have already observed that plants suffered from Fe deficiency could form transfer cells and subapical root hairs from root epidermal cells and even develop cluster roots, as well as swollen apical root tips (Schmidt et al., 2000; Schikora and Schmidt, 2001, 2002). These morphological changes have been believed to increase the absorption surface area, evolutionally facilitate the growth of plants under Fe starvation. Along with these findings, ethylene has always been reported to be involved in these morphological alterations under Fe deficiency, which is revealed by the use of ethylene inhibitors and precursors, as well as the use of ethylene mutants (Schmidt et al., 2000; Curie et al., 2001; Garcia et al., 2015). Subapical root hairs, transfer cells, and cluster roots have been found to be inhibited by the addition of ethylene inhibitors to the Fe-deficient roots, while an increase of the ethylene level will promote their formation even under Fe-sufficient conditions (Schmidt et al., 2000; Garcia et al., 2015). Similarly, the formation of subapical root hairs was blocked in the ethylene insensitive mutants *etr1* and *ein2* in *Arabidopsis, etr1* in the soybean and *sickle* in the *Medicago truncatula* both under Fe limiting and the addition of ethylene precursors (Garcia et al., 2015). However, the boosted ferric reductase activity and the expression of Fe acquisition genes are not affected in these mutants under Fe starvation, implying that morphological and physiological responses are carried out by different regulatory mechanism (O'rourke et al., 2007a,b, 2009). How ethylene exerts its distinct roles under Fe deficiency remains to be further explored.

## Transcriptomics of Fe dificiency and the invovlement of ethylene in strategy I plants

Since 2001, toward the genome-wide understanding of plant response to Fe deficiency, transcriptome profiling studies, using custom-made or commercial-based microarrays and next-generation sequencing-based techniques (RNA-seq), have been carried out in a broad range of plant species, including model strategy I plant Arabidopsis, Medicago, soybean, as well as Strategy II plant rice and others (Thimm et al., 2001; Wang et al., 2003; Colangelo and Guerinot, 2004; Besson-Bard et al., 2009; Buckhout et al., 2009; Garcia et al., 2010; Sivitz et al., 2012; Zamboni et al., 2012; Li et al., 2013; Lan et al., 2013b; Rodriguez-Celma et al., 2013b; Bashir et al., 2014; Pan et al., 2015; Mai et al., 2016). Most of these omic studies have been carried out in the wild type plants either in Fe-deficient roots or shoots, also in the whole seedlings, while few of them are performed either in the ethylene mutants under Fe deficiency (Bauer and Blondet, 2011) or under Fe deficiency with ethylene inhibitors (Garcia et al., 2010). Nevertheless, a suit of Fe deficiency-regulated genes was revealed to be ethylene responsive by omic studies (Garcia et al., 2010; Bauer and Blondet, 2011; Lingam et al., 2011), and their expression depending on ethylene was further confirmed by reverse transcription-PCR (RT-PCR) as well as enzyme activity determination (Lucena et al., 2006; Garcia et al., 2010).

Although transcriptomic studies have identified hundreds and thousands of Fe-responsive genes, the overlapping genes among different studies are not high, probably due to the variations in growth conditions, sampling time and handling, differences in the experimental design, or combinations of these factors. Nevertheless, some novel regulators, such as FIT, MYB72, MYB10, and POPEYE, controlling plant response to Fe deficiency have been identified by omic approaches and further confirmed by genetic studies (Colangelo and Guerinot, 2004; Long et al., 2010; Palmer et al., 2013). Moreover, each of these studies has uncovered, more or less, a subset of Fe-responsive genes, which are also regulated by ethylene. To identify such group of genes that are robustly regulated by both Fe deficiency and ethylene across a wide range of conditions, publicly accessible transcriptomic data were first surveyed to define Fe-responsive core genes, followed by mining those genes that are also regulated by ethylene from these Fe-responsive core genes. Since most of extensive transcriptomic studies focused on the model plant Arabidopsis, the core Fe-responsive genes are depicted here for Arabidopsis. Three microarray studies relying on commercially available Affymetrix ATH1 GeneChips (Dinneny et al., 2008; Yang et al., 2010; Mai et al., 2016) and one RNA-seq data set (Lan et al., 2013b) have been chosen for this attempt. The reason for choosing these studies is below: (1) the plants used in all these studies were similar age; (2) all these studies provided both up- and down-regulated genes upon Fe deficiency; (3) all these studies provided differentially expressed genes in wild type plants.

**Figure 1:**
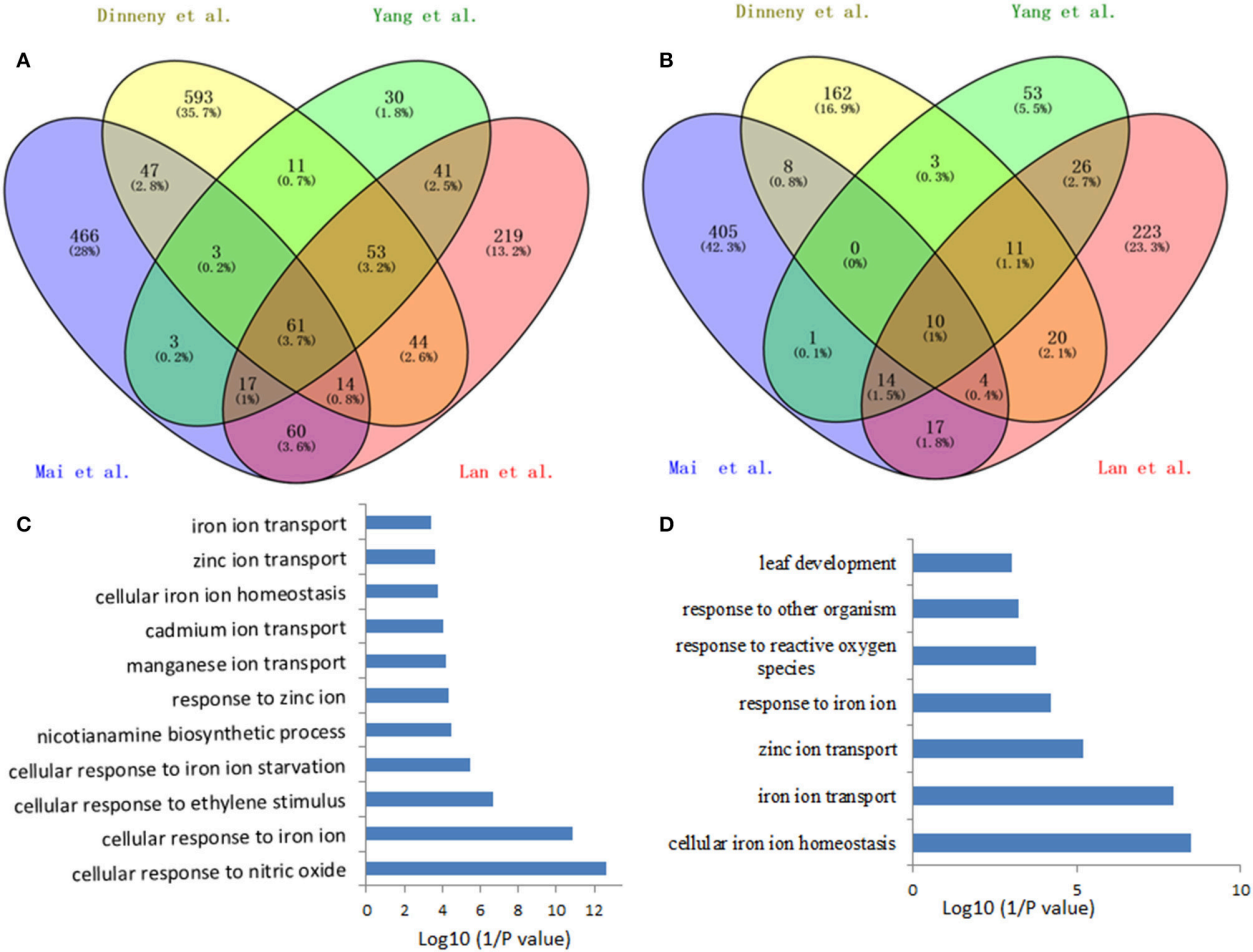
**Venn diagram of the overlap of the iron deficiency response genes among the data sets for defining the conserved iron starvation response genes. (A)** upregulated genes; **(B)** downregulated genes; **(C,D)** Gene ontology enrichment analysis (biological process, *P* < 0.001) of the overlap genes, 61 and 10, from the up- and down-regulated genes under iron starvation among the four data sets, respectively.

Such analysis results in an overlap of 71 differentially expressed genes (fold-change ≥ 1.5 and *P* < 0.05) from the four data sets, herein named Fe deficiency-response core genes, with 61 being up-regulated and 10 down-regulated (Figure 1; Table 1). Although no ethylene-responsive gene is found in the down-regulated core genes, a subset of seven ethylene-responsive genes, including the transcription factor genes *bHLH39* (Yuan et al., 2008; Garcia et al., 2010) and *MYB72* (Zamioudis et al., 2014), Fe transporter gene *IRT1* (Garcia et al., 2013), and Fe homeostasis gene *NAS1* (Garcia et al., 2010), methylthioribose kinase gene *MTK* (Burstenbinder et al., 2007; Garcia et al., 2011), as well as other two genes AT3G12900 (Garcia et al., 2010) and AT5G38820 (Garcia et al., 2010) encoding proteins with unidentified functions, is identified within the up-regulated core genes (Table 1). Although the number of identified genes, whose expression is affected by ethylene, is small in the Fe responsive core genes, most of them have very significant roles in Fe acquisition and homeostasis. By forming heterodimers with FIT, bHLH39 controls the expression of subsets of Fe responsive genes (Yuan et al., 2008), while MYB72 directly regulates the expression of beta-Glucosidase BGLU42, a key player of rhizobacteria-induced systemic resistance (Zamioudis et al., 2014), and is required for plant growth under Fe deficiency (Palmer et al., 2013). Iron transporter IRT1 is the major transporter absorbing Fe^2+^ from soil, required for plant survival (Vert et al., 2002). It has been recognized that the nicotianamine (NA) is a key chelator of Fe^2+^, involved in the phloem-based transport of Fe to sink organs such as young leaves and seeds, thus mediating Fe homeostasis; while NA is synthesized by NA synthase (NAS) from S-adenosylmethionine (SAM), underlying the importance of controlling the expression of NAS genes including NAS1 gene (Schuler et al., 2012). *MTK*, an enzyme of the “Yang cycle” maintaining methionine (Met) recycling for ethylene synthesis, is encoded by single gene in Arabidopsis genome, indicating the importance to tightly control its expression (Burstenbinder et al., 2007). Expression of *MTK* was induced by both ethylene and Fe deficiency, suggesting proper ethylene production might be crucial for plant fitness under Fe shortage conditions. Gene AT3G12900 encodes a protein belonging to the 2OG-Fe(II) oxygenase family; proteins in this family is generally considered to possess oxidoreductase activity catalysing the 2-oxoglutarate- and Fe(II)-dependent oxidation of an organic substrate using a dioxygen molecule. Previous study showed that the ethylene synthesis protein ACC oxidase also belongs to 2OG-Fe(II) oxygenase superfamily (Aravind and Koonin, 2001). Therefore, it is reasonable to assume that this gene might be involved in the ethylene production under Fe deficiency, although its exact biological functions remain to be verified. Omic studies thus revealed the involvement of ethylene in the Fe deficiency response.

**Table 1:** **Iron deficiency regulated conserved genes in *Arabidopsis***.

**AGI^a^**	**Name and annotation**	**Dinneny et al., 2008**				**Yang et al., 2010**	**Lan et al., 2013b**	**Mai et al., 2016**
		**−Fe**				**−Fe 3d**	**−Fe 3d**	**−Fe 6 d**
		**12h**	**24 h**	**48 h**	**72 h**			
		**Roots**				**Roots**	**Roots**	**Seedlings**
**Fold-changes (−Fe/+Fe)**								
**UP-REGULATED CORE GENES UNDER Fe DEFICIENCY**								
**Glycolysis**								
AT1G53310	PPC1, phosphoenolpyruvate carboxylase	1.0	2.2	2.6	2.1	2.2	2.4	1.6
**Cell wall**								
AT1G09790	Phytochelatin synthetase-related	1.2	3.3	5.9	5.3	8.4	53.4	1.9
AT4G02330	Pectinesterase family protein	1.9	11.0	21.3	21.3	10.3	4.8	1.9
**Amino acid metabolism**								
AT1G49820	MTK, 5-methylthioribose kinase, involved in methionine cycle	1.1	2.8	3.6	3.3	2.5	2.7	1.7
**Metal handling**								
AT1G23020	FRO3, ferric chelate reductase 3	1.5	5.6	6.4	5.1	8.4	8.0	3.9
AT1G56430	NAS4, nicotianamine synthase 4	1.8	6.8	14.6	16.3	5.0	5.0	2.9
AT5G04950	NAS1, nicotianamine synthase 1	1.2	3.7	4.9	5.0	3.1	4.4	2.0
**Secondary metabolism**								
AT3G21240	4CL2, 4-coumarate:CoA ligase 2	1.2	3.5	5.6	5.7	3.9	3.6	1.6
AT3G50740	UGT72E1, UDPG:coniferyl alcohol glucosyltransferase	1.1	3.8	6.0	5.9	4.2	8.5	2.2
**Hormone metabolism**								
AT3G12900	Oxidoreductase, 2OG-Fe(II) oxygenase family protein	1.7	11.4	33.0	36.6	97.2	546.9	14.7
**Stress**								
AT1G09560	GLP5, GERMIN-LIKE PROTEIN 5	1.1	2.4	2.3	2.1	3.0	7.7	3.7
AT2G42750	DNAJ heat shock N-terminal domain-containing protein	1.3	3.0	4.8	4.1	2.0	1.9	2.2
AT3G48450	Nitrate-responsive NOI protein, putative	1.4	3.9	9.5	8.6	8.1	5.1	2.0
AT5G45070	PP2-A8, PHLOEM PROTEIN 2-A8	0.8	2.6	6.3	5.8	6.0	8.4	3.7
**Misc**								
AT4G31940	CYP82C4, cytochrome P450 enzyme	2.1	6.6	9.3	10.8	71.8	188.0	48.6
AT5G02780	In2-1 protein, the lambda family of glutathione transferases	2.6	9.7	12.9	11.4	30.7	54.7	19.6
**RNA**								
AT1G56160	MYB72, R2R3 transcription factor	1.3	4.2	6.5	6.8	86.1	∞	7.5
AT3G12820	MYB10, R2R3 transcription factor	1.3	3.5	6.2	6.6	24.3	24.6	4.1
AT3G18290	EMB2454, BRUTUS (BTS), a putative E3 ligase protein	1.5	4.8	7.7	8.4	2.7	2.5	2.5
AT3G47640	POPEYE (PYE), a bHLH transcription factor	1.2	2.5	3.6	3.7	2.6	2.2	3.2
AT3G56980	ORG3, bHLH39	1.6	4.6	6.0	5.6	26.4	31.8	10.6
AT5G04150	BHLH101	1.6	5.8	7.0	6.7	13.9	18.0	9.0
**DNA**								
AT3G13610	F6′H1, Fe(II)-, and 2-oxoglutarate-dependent dioxygenase	1.5	4.6	6.4	6.2	5.4	10.3	2.8
**Protein**								
AT1G18910	Zinc finger (C3HC4-type RING finger) family protein	1.1	3.0	5.2	5.0	3.3	3.3	1.6
AT1G60610	Protein binding / zinc ion binding	1.3	2.2	3.4	2.8	2.3	2.0	1.8
AT1G77280	Protein kinase family protein	1.1	2.6	4.2	4.1	3.8	4.1	1.6
AT4G09110	Zinc finger (C3HC4-type RING finger) family protein	1.0	1.5	2.5	2.3	3.6	19.6	6.0
AT4G10510	Subtilase family protein	1.1	1.5	4.3	3.7	5.8	7.1	3.6
AT4G26470	Calcium ion binding	1.1	2.0	3.3	2.8	1.8	1.6	2.1
AT5G53450	ORG1, OBP3-RESPONSIVE GENE 1	1.4	5.8	7.4	6.8	5.4	5.2	5.9
**Signaling**								
AT1G16150	WAKL4, WAK-like receptor-like kinase	1.2	2.1	3.6	3.0	2.4	2.7	1.6
AT1G34760	GRF11, GENERAL REGULATORY FACTOR 11	1.2	3.7	5.1	4.1	9.4	14.7	2.7
AT4G29900	ACA10, AUTOINHIBITED CA(2+)-ATPASE 10	1.5	3.2	4.4	3.8	2.3	1.9	1.6
**Transport**								
AT3G46900	COPT2, COPPER TRANSPORTER 2	1.6	5.5	6.5	6.8	15.7	42.9	8.7
AT3G53480	ABCG37/PDR9, ATP-BINDING CASSETTE G37/ PLEIOTROPIC DRUG RESISTANCE 9	0.9	2.5	3.2	2.8	3.5	5.3	2.1
AT3G58060	MTP8, Mn transporter	1.2	3.3	6.5	6.2	22.4	54.9	1.7
AT3G58810	MTPA2, METAL TOLERANCE PROTEIN 3	1.3	3.8	4.8	4.3	9.7	17.1	3.4
AT4G16370	OPT3, OLIGOPEPTIDE TRANSPORTER	1.9	6.5	9.4	10.3	7.6	7.0	2.6
AT4G19690	IRT1, IRON-REGULATED TRANSPORTER 1	1.4	2.4	2.3	2.3	21.8	57.1	38.6
AT5G01490	CAX4, CATION EXCHANGER 4	1.1	2.2	3.6	3.4	2.0	2.6	1.7
AT5G03570	IREG2, IRON REGULATED 2	1.2	4.5	7.4	6.8	6.7	7.7	3.5
AT5G13740	ZIF1, ZINC INDUCED FACILITATOR 1	1.5	5.6	8.7	8.2	2.6	2.8	2.8
AT5G38820	Amino acid transporter family protein	1.4	3.8	5.6	6.0	17.0	49.6	3.5
AT5G67330	NRAMP4, ARABIDOPSIS THALIANA NATURAL RESISTANCE ASSOCIATED MACROPHAGE PROTEIN 4	1.5	4.3	6.3	6.6	2.2	2.4	1.7
**Not assigned**								
AT1G12030	Unknown protein	1.0	1.4	5.3	6.4	2.4	21.5	8.9
AT1G22930	T-complex protein 11	1.0	2.6	3.6	3.3	1.9	2.2	1.6
AT1G47400	Unknown protein	1.8	5.8	11.6	11.5	15.7	18.5	16.3
AT1G49000	Unknown protein	1.2	2.7	5.4	4.8	6.1	6.7	2.1
AT1G73120	Unknown protein	1.4	3.3	3.1	2.4	8.4	7.3	8.8
AT1G74770	Unknown protein	1.1	2.8	4.7	3.9	5.3	6.3	3.6
AT2G46750	FAD-binding domain-containing protein	1.6	3.4	4.6	3.4	2.8	2.7	3.4
AT3G06890	Unknown protein	1.3	2.8	4.2	3.8	3.6	3.3	2.0
AT3G07720	Kelch repeat-containing protein	1.9	5.5	6.2	5.2	11.9	23.0	7.9
AT3G18560	Unknown protein	1.0	2.7	3.9	4.4	4.0	3.6	1.9
AT3G56360	Unknown protein	1.2	2.4	2.6	2.7	2.3	2.3	2.7
AT3G61410	Unknown protein	1.1	2.2	3.0	2.6	4.3	6.0	2.4
AT3G61930	Unknown protein	2.4	7.3	14.0	13.2	24.7	24.3	4.9
AT5G02580	Unknown protein	1.1	3.1	5.2	5.8	2.6	1.8	2.1
AT5G05250	Unknown protein	1.4	3.2	4.5	4.7	6.1	6.5	4.7
AT5G61250	Glycosyl hydrolase family 79 N-terminal domain-containing protein	1.5	3.3	4.0	3.3	2.6	2.6	1.7
AT5G67370	Unknown protein	1.5	5.7	11.2	12.1	7.4	9.8	2.8
**DOWN-REGULATED CORE GENES UNDER Fe DEFICIENCY**								
**Metal handling**								
AT3G56090	FER3, FERRITIN 3	1.0	0.9	0.6	0.5	0.5	0.5	0.5
AT5G01600	FER1, FERRITIN 1	0.7	0.5	0.4	0.4	0.2	0.2	0.4
**Secondary metabolism**								
AT2G37130	Peroxidase 21	1.1	0.9	0.2	0.1	0.4	0.5	0.6
**Redox**								
AT4G08390	SAPX, STROMAL ASCORBATE PEROXIDASE	0.9	0.7	0.4	0.3	0.5	0.5	0.6
**Misc**								
AT3G09220	LAC7, LACCASE 7	1.2	1.3	0.3	0.2	0.3	0.3	0.3
**Protein**								
AT4G04770	ABC1, ARABIDOPSIS THALIANA NUCLEOSOME ASSEMBLY PROTEIN 1	0.9	0.7	0.4	0.4	0.4	0.4	0.6
**Transport**								
AT1G60960	IRT3, IRON REGULATED TRANSPORTER 3	0.8	0.6	0.3	0.3	0.4	0.5	0.3
AT2G32270	ZIP3, ZINC TRANSPORTER 3 PRECURSOR	0.5	0.4	0.2	0.2	0.4	0.5	0.3
**Not assigned**								
AT1G68650	Unknown protein	0.9	0.7	0.5	0.5	0.4	0.4	0.4
AT2G36885	Unknown protein	0.9	0.6	0.4	0.4	0.4	0.3	0.4

a *Underlined genes were also regulated by ethylene*.

Among the up-regulated core genes, those genes encoding proteins with unknown functions represent the largest group, indicating that molecular mechanisms of Fe deficiency responses remain incomplete and potentially novel players could be discovered in the future study. In this group, none of them is ethylene responsive. Of particular interest are three highly induced genes AT1G12030, AT1G47400, and AT5G05250, which are tightly co-expressed with several well-studied Fe-related genes such as POPEYE (Long et al., 2010), NAS4 (Koen et al., 2013), OPT3 (Zhai et al., 2014), bHLH101 (Sivitz et al., 2012), etc. POPEYE has been reported to be required for plant growth and development under Fe-deficient conditions, probably through affecting cellular Fe homeostasis by directly regulating the expression of known Fe homeostasis genes such as *NSA4* and *FRO3* (Long et al., 2010), while OPT3 has been verified to be a phloem-specific Fe transporter, essential for systemic Fe signaling and redistribution of Fe in Arabidopsis (Mendoza-Cozatl et al., 2014; Zhai et al., 2014). Thus, it is reasonable to assume that these co-expressed presently functional unknown genes could play important roles in Fe homeostasis or in Fe distribution intracellularly and/or intercellularly. Other three genes AT3G07720, AT3G61930, and AT5G67370 are also highly induced by Fe deficiency and this induction occurs in the early treatment and lasts for 3 days, suggesting that these genes might be involved in the early Fe deficiency response and their biological functions are worthy of further study. To meet the cellular demand of Fe under Fe deficiency, plants have evolutionally developed a complex mechanism to cope with Fe starvation, including the release of vacuolar Fe via transporter (Lanquar et al., 2005), increasing the rhizosphere available Fe via ABCG37/PDR9 (Rodriguez-Celma et al., 2013b; Fourcroy et al., 2014), as well as an enhanced uptake of Fe into the cell through the induction of IRT1 (Vert et al., 2002). Genes encode these transporters were observed to be induced by Fe deficiency in all studies, with IRT1 being ethylene responsive (Garcia et al., 2013). However, the non-specific transport character of IRT1 allows the transport of other essential and non-essential metals besides Fe, which results in an enhanced concentration of these metals. To avoid toxicity of these byproducts, an array of genes encoding transporters were observed to be induced under Fe deficiency, including copper transporter (Perea-Garcia et al., 2013), Zinc transporter (Haydon et al., 2012), manganese transporter (Arrivault et al., 2006), and other cation transporters, which could sequester these ions into vacuoles. These results indicate that plants have evolved an excellent system to monitor the cellular ions, maintaining the homeostasis of required ions, and avoiding the toxicity of “unwanted” ions, under Fe deficiency and maybe upon other nutritional disorder. In addition, genes encode transporters OPT3 (Zhai et al., 2014) and AtIREG2 (Schaaf et al., 2006), which are involved in the intercellular Fe distribution, are robustly induced by Fe shortage, underlying the importance of Fe distribution upon Fe deficiency. High induction was also observed for the gene AT5G38820 whose expression is also controlled by ethylene, which encodes putatively an amino acid transporter. By now, it remains unclear what is the biological function of this transporter. Is it possible to transport Met under Fe deficiency? Indeed, as mentioned above, the gene encoding MTK was robustly up-regulated by Fe deficiency (Dinneny et al., 2008; Garcia et al., 2010; Yang et al., 2010; Lan et al., 2013b; Mai et al., 2016). MTK is a key kinase of Yang-cycle involved in the Met salvage by phosphorylating methylthioribose (MTR). Phosphorylated MTR will finally be converted to Met by serials of biochemical reactions. Met is then converted to SAM which is used to synthesize ethylene, polyamines, NA, as well as phytosiderophores in Strategy II species. Although neither ACC synthase (ACS) gene nor ACC oxidase (ACO) gene was found within the Fe deficiency response core genes by transcriptomics, transcriptional expression changes of these genes do have been monitored by RT-PCR and their encoded proteins were differentially accumulated upon Fe deficiency (Garcia et al., 2010; Ye et al., 2015), which is consistent with the increase of ethylene production under Fe deficiency (Lucena et al., 2015). By contrast, two of three genes associated with metal handling encode NAS were up-regulated by Fe deficiency with *NAS1* and *NAS2* being ethylene responsive (Garcia et al., 2010); while NA, which is synthesized by NAS, is a key chelator of Fe^2+^, involved in the phloem-based transport of Fe to sink organs (Schuler et al., 2012). These results suggest that ethylene and NA are both important for the regulation of Fe deficiency responses. A group of genes encode transcription factors were strongly induced among the core genes, with MYB72 and MYB10 being shown to be functional redundancy but crucial for plant growth under iron-limiting conditions (Palmer et al., 2013). In addition, several genes encoding regulatory proteins, such as protein kinases, involved in posttranslational protein modification, RING domain-containing Zinc finger family proteins, associated with protein degradation and other zinc/calcium binding proteins, have been shown upregulated among the core genes, although most of them remain functionally unclear. Besides, three genes encoding proteins involved in the signaling were identified in the core, with the general regulatory factor 11 (GRF11) being confirmed to be a key downstream player of nitric oxide to regulate Fe acquisition (Yang et al., 2013). Surprisingly, only two genes encoding proteins involved in cell wall process are presented in the core, despite the pronounced alternations in the root morphology. Similarly, one gene encoding an isoform of 4-coumarate: CoA ligase (4CL2) involved in the last step of the general phenylpropanoid pathway was significantly induced by Fe starvation. In addition, several stress-response genes are also represented in the upregulated core genes.

In total, 10 genes were observed down-regulated in the core genes, but none of them is ethylene responsive. In Arabidopsis genome, four genes encoding FERRITINs (FER), functioning as ferric iron binding and participating in the cellular iron ion homeostasis, are essential to protect cells against oxidative damage and flowering (Sudre et al., 2013). Two genes encoding FER3 and FER1 were identified to be robustly down-regulated in the core genes. In addition, two genes encoding a plasma membrane localized zinc/iron transporter IRT3 (Lin et al., 2009) and a Zn^2+^ transporter ZIP3 (Yang et al., 2010) were down-regulated under Fe deficiency in order to decrease the uptake of zinc ion under Fe starvation, avoiding toxicity of excess zinc. Gene AT2G37130, which encodes a peroxidase and is associated with defense response to fungus, hydrogen peroxide catabolic process, oxidation-reduction process, and oxidative stress response, was identified in the core with less-explored. Similarly, three genes encoding a chloroplastic stromal ascorbate peroxidase SAPX (Maruta et al., 2010), a laccase LAC7 (Turlapati et al., 2011), and an iron-stimulated ATPase ABC1 (Moller et al., 2001), respectively, have been identified in the down-regulated core genes. Ascorbate peroxidases are enzymes possessing hydroquinone:oxygen oxidoreductase activity that can scavenge hydrogen peroxide in plant cells, and laccase has oxidoreductase activity capable of oxidizing metal ions, involving lignin catabolic process and oxidation-reduction process, while ABC1 belongs to the member of the NAP subfamily of ABC transporters involved in Fe-S cluster assembly (Xu and Moller, 2004; Xu et al., 2005). Thus, similar to SufB, AtABC1 is associated with the regulation of iron homeostasis (Xu and Moller, 2004; Xu et al., 2005). Notably, two genes AT1G68650 and AT2G36885 encoding proteins with unknown functions were observed to be down-regulated more than two-fold and await further study.

## CO-expression network of Fe/ethylen response core genes

**Figure 2:**
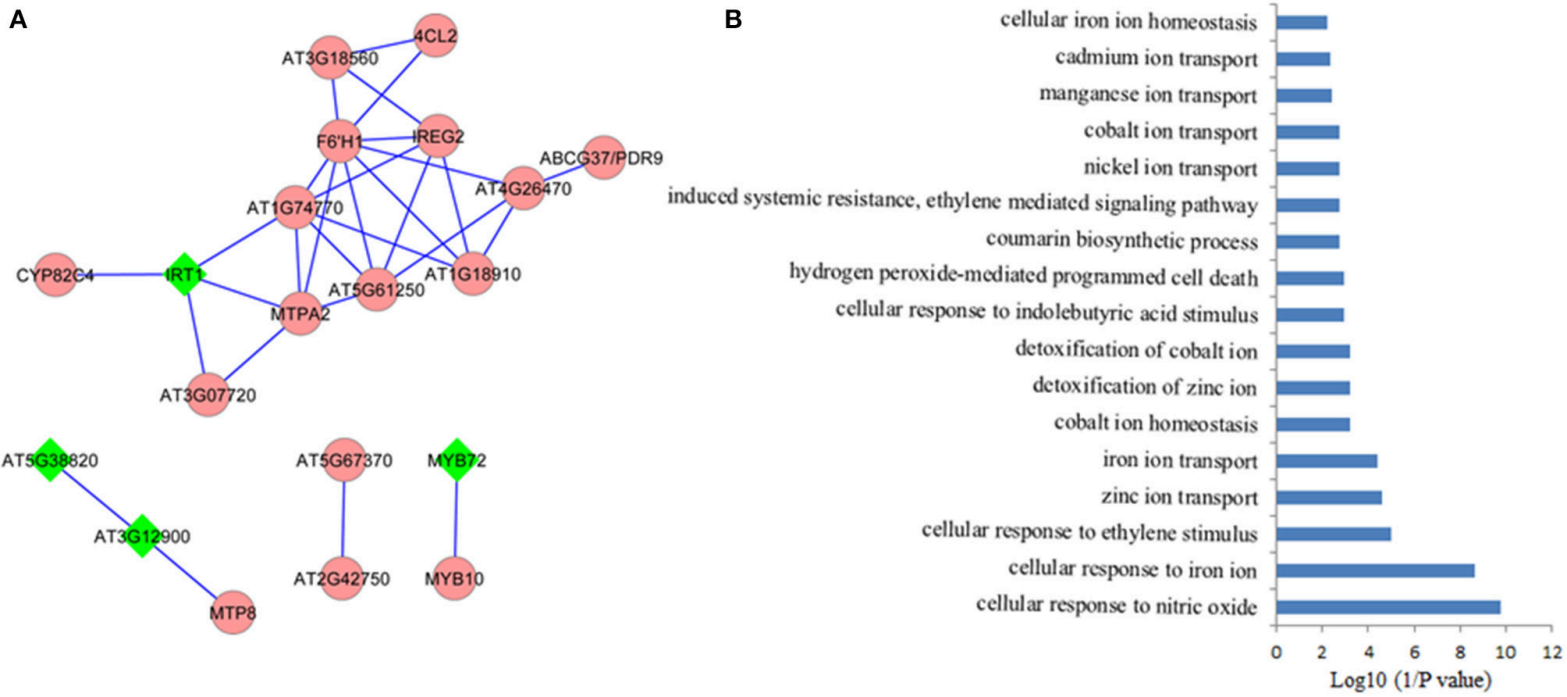
**Coexpression network of the conserved iron deficiency induced genes. (A)** Pair-wise coexpression relationships were calculated with the MACCU toolbox as described by Lin et al. (2011) using a Pearson coefficient cut-off of *P* ≧ 0.7. Nodes in diamond filled in green color indicate ethylene regulated genes; **(B)** Gene ontology (GO) enrichment analysis (biological process, *P* < 0.01) of the genes from the network in **(A)**.

It is assumed that genes showing similar expression patterns under various conditions have a high possibility to exert similar functions. Co-expression network can help to predicate the functions of those genes, which remain unclear, from those well-studied genes in the network via the “guilt-by-association” paradigm. In addition, co-expression network might also work in identifying novel functions of the well-known “old” genes under certain conditions. Co-expression network of the 71 Fe deficiency response core genes was generated using MACCU program from 300 public microarrays restricted to roots with a Pearson correlation coefficient greater or equal to 0.7, a threshold frequently used to create compression networks of relatively high stringency (Lin et al., 2011). This procedure yielded a co-expression network containing 20 core genes, subdivided into four subclusters (Figure 2A). Unexpectedly, none of the down-regulated core genes was co-expressed in the network, suggesting that these down-regulated core genes might be associated with diverse biological processes. Four out of 20 genes (nodes) in the co-expression network were also ethylene responsive (nodes in diamond shape) and were distributed in the three subclusters (Figure 2B). In the largest subcluster, several genes encoding transporters, such as IRT1(Vert et al., 2002), ABCG37/PDR9(Rodriguez-Celma et al., 2013b), IREG2 (Schaaf et al., 2006), and MTPA2 (Arrivault et al., 2006) associated with Fe acquisition and distribution, as well as the sequestration of Zn^2+^ into vacuoles, have been identified as strongly co-expressed, particularly the direct connectivity of IRT1 and MTPA2, indicating that avoiding toxicity of excess Zn might be equally important to Fe absorption and distribution under Fe deficiency. Among the genes with the highest connectivity (largest amount of edges) was the gene AT3G13610 encoding a Fe (II)- and 2-oxoglutarate-dependent dioxygenase family gene F6'H1 (Rodriguez-Celma et al., 2013b). Mutations in this gene compromise iron uptake and the production of fluorescent phenolics involved in Fe uptake. The second largest subcluster comprises three genes. AT3G58060 encodes a tonoplast localized member of CDF family of cation transporters MTP8 (Eroglu et al., 2016), which functions as a manganese (Mn) transporter. MTP8 transports Mn ion into root vacuoles of iron-deficient plants and thereby avoids toxicity of excess Mn ion in Fe deficiency-roots. AT3G12900, an ethylene response gene (Garcia et al., 2010), directly connected with *MTP8*, is strongly induced by Fe deficiency, which encodes a 2-oxoglutarate (2OG) and Fe (II)-dependent oxygenase superfamily protein putatively assumed to be involved in hormone metabolism by MapMan analysis. The gene co-expressed with AT3G12900 is AT5G38820, whose expression is also regulated by ethylene (Garcia et al., 2010), encoding a putative amino acid transporter with no known function yet. Each of the other two subclusters contains only two genes, with one cluster comprising MYB72 and MYB10 (Palmer et al., 2013), and the other one being composed of two functional unknown genes. *MYB72*, an ethylene responsive gene (Garcia et al., 2010), and *MYB10* have been confirmed to be crucial for plant growth under Fe shortages (Palmer et al., 2013; Zamioudis et al., 2014). AT5G67370 and AT2G42750 were directly connected, comprising a small network. AT5G67370 encodes a protein of unknown function, while mutations in this gene shows a stronger growth defect compared to wild type in low Fe (1 μM Fe) conditions (Urzica et al., 2012). AT2G42750 encodes a DNAJ heat shock N-terminal domain-containing protein; gene ontology (GO) analysis shows the protein encoded by this gene can function in unfolded protein and heat shock protein binding, involving electron carrier activity and iron ion binding. In summary, several genes encoding proteins with unknown functions were observed to be directly connected to these well-studied genes, indicating that these functional unknown genes might be crucial for Fe homeostasis under Fe deficiency. Co-expression network thus filter out prioritized genes for follow-up research from large data sets derived from high-throughput omic approaches.

## Proteomes of Fe deficiecny in plants

Although high-throughput transcriptomic studies have provided a global view of gene expression changes, surveys at the transcript level often could not directly estimate the abundance and functions of the encoded proteins, the ultimate players of biological function, due to alternative RNA splicing and complex posttranslational modifications such as phosphorylation, glycosylation, methylation, ubiquitination, and so on (Lan et al., 2012a, 2013a; Marmiroli et al., 2015; Dong et al., 2016). These various modifications thus change protein localization, stability, interactions, and functions, leading to much more complex of proteomics than that of transcriptomics. Therefore, a global view of protein changes both in abundance and isoforms can only be relatively precisely estimated and identified by proteomics methods.

With the advances in Mass spectrometry techniques coupled with powerful computational algorithms, a wealth of knowledge has arisen on the protein changes upon various stresses including Fe deficiency (Lopez-Millan et al., 2013). Overall, compared to the extensive transcriptomic investigation upon Fe deficiency, knowledge on the changes in the proteome in response to Fe deficiency has been still largely limited, particularly in Strategy II species (Lopez-Millan et al., 2013). So far, only one study on proteome profiling in Fe-deficient rice roots and shoots (Chen et al., 2015) and two in maize, with one in Fe-deficient root hairs (Li et al., 2015) and the other in roots (Hopff et al., 2013), have been carried out to investigate the global protein changes or the alterations in the plasma membrane proteome, by means of two-dimensional electrophoresis (2-DE), or 1-DE coupled with matrix-assisted laser desorption/ionization time of flight mass spectrometry (MALDI-TOF/MS) or LC-MS/MS. By contrast, most of the proteomic studies upon Fe deficiency have been performed in Strategy I species, including *Beta vulgaris* (Andaluz et al., 2006; Rellan-Alvarez et al., 2010; Gutierrez-Carbonell et al., 2016), Tomato (*Solanum lycopersicum* L.) (Brumbarova et al., 2008; Genannt Bonsmann et al., 2008; Muneer and Jeong, 2015), cucumber (*Cucumis sativus*) (Donnini et al., 2010; Li and Schmidt, 2010; Vigani et al., 2017), pea (*Pisum sativum* L.) (Meisrimler et al., 2011, 2016), Prunus hybrid GF 677 rootstock (*P. dulcis* × *P. persica*) (Rodriguez-Celma et al., 2013a), *Lupinus texensis* (Lattanzio et al., 2013), *Hyoscyamus albus* (Khandakar et al., 2013), *Medicago truncatula* (Rodriguez-Celma et al., 2011, 2016), citrus rootstocks (Muccilli et al., 2013), *Brassica napu* (Gutierrez-Carbonell et al., 2015), *Populus cathayana* (Zhang S. et al., 2016), and Arabidopsis (Laganowsky et al., 2009; Lan et al., 2011, 2012b; Mai et al., 2015; Pan et al., 2015; Zargar et al., 2015a,b). Most of the protein profiling studies were focused on the global protein changes in the whole roots/shoots, and few of them investigated proteome of the specific plant parts such as root hairs (Li et al., 2015), cellular compartments including root plasma membrane (Hopff et al., 2013), thylakoid membranes (Andaluz et al., 2006), and shoot microsomal fragments (Zargar et al., 2015b), as well as phloem saps (Lattanzio et al., 2013; Gutierrez-Carbonell et al., 2015).

Several features of Fe-related proteomics should be noticed. First, the focuses of the proteomic studies under Fe limiting conditions by now are still the determination and quantification of changed proteins. So far, only one proteomic study has explored the alterations of protein posttranslational modification (PTM), and this study does have uncovered new layer of regulation in gene activity in response to Fe deficiency (Lan et al., 2012b), suggesting that PTM proteomic studies should be emphasized in the future. Second, until 2011, a gel-based approach (particularly 2DE) is the most commonly employed approach to explore the changes in protein abundance upon Fe deficiency. Overall, the number of proteins that are identified to be changed in abundance by 2DE-based proteomics is generally not high, often ~50 by mean, which is irrelevant to the gel size (Lopez-Millan et al., 2013). Moreover, most of the identified proteins are highly abundant soluble proteins which associated with various metabolisms, with few proteins involved in transporting, signaling and regulation being uncovered. Furthermore, exact quantification of differentially expressed proteins has proven difficult with this approach. Since 2011, high-throughput proteomic analyses have been carried out by means of iTRAQ (Isobaric Tag for Relative and Absolute Quantification) based LC-MS (Lan et al., 2011; Zargar et al., 2013, 2015a,b; Pan et al., 2015; Zhang S. et al., 2016) and label-free LC-MS (Li et al., 2015; Meisrimler et al., 2016), although 2DE is still in use. These approaches have proven powerful to increase not only the numbers of differentially accumulated proteins but also the types of proteins, which provide much more knowledge of plant response to Fe deficiency. Third, although each of the proteomic studies can yield more or less differentially accumulated proteins, and those proteins, with the help of bioinformatics, can be further assigned to one or more metabolic pathways to a certain extent, the direct comparison of proteomes both among plant species, and within the same species has proven not straightforward, due to various differences in each proteomics study, from growth conditions, sampling time and handling, experimental design, types of spectrometry and computational software, stringency of searching parameters, to richness of genomic information.

## Proteomics reveal that S-adenosyl-methionine synthesis is one of the central metabolic processes upon Fe deficiency

**Figure 3:**
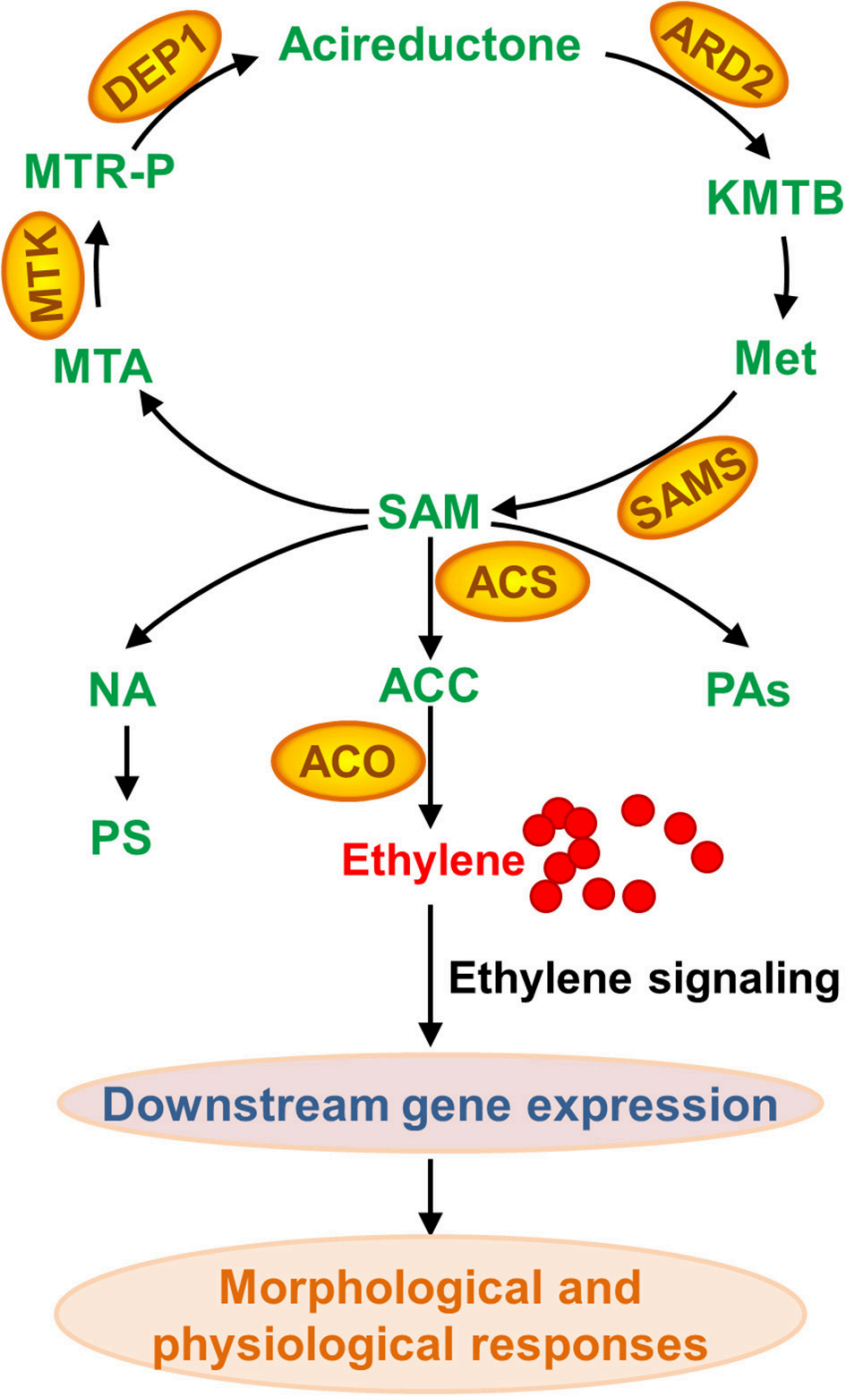
**Methionine (Met) salvage cycle and ethylene, nicotianamine (NA), phtosiderophore (PS), and polyamine (PA) synthesis associated with proteins that are differentially accumulated upon Fe deficiency**. As a central metabolite, SAM (S-adenosyl-methionine) is synthesized by SAMS (S-adenosylmethionine synthetase) from Met. Followed, SAM can be converted to MTA (methylthioadenosine) and ACC (aminocyclopropane-1-carboxylate) that is further converted to ethylene. SAM is also a precursor of PA and NA, which is converted to PS in Strategy II plant species. KMTB, 2-keto-4-methylthiobutyrate; MTR-P, methylthioribose phosphate; ARD, acireductone dioxygenase; ACS, ACC synthase; MTK, 5-methylthioribose kinase; DEP1, EHYDRATASE-ENOLASE-PHOSPHATASE-COMPLEX1; ACO, ACC oxidase.

Although nearly no protein with changes in abundance upon Fe deficiency has been uncovered across a wide range of plant species, a comprehensive comparison of these studies indeed has revealed some common elements in proteome under Fe limitation. In brief, proteins associated with “oxidative stress and defense,” “C metabolism,” “N metabolism,” “cell wall,” “secondary metabolism, particularly the phenylpropanoid metabolism,” “energy and ATP-coupled transport processes,” and “protein metabolism” have been identified as differentially accumulated proteins among plant species, which is well-summarized in an excellent review published in 2013 (Lopez-Millan et al., 2013). Update of that review is out of the scope of present one. By contrast, this review will focus on the changed proteins associated with methionine (Met) salvage cycle and ethylene synthesis under Fe shortage (Figure 3). Indeed, as shown in Table S1, many of the proteomic studies have uncovered that S-adenosyl-methionine (SAM), a precursor for ethylene production, is a central metabolite under Fe deficiency in multiple plant species (Li et al., 2008; Donnini et al., 2010; Li and Schmidt, 2010; Lan et al., 2011; Rodriguez-Celma et al., 2011; Gutierrez-Carbonell et al., 2015; Pan et al., 2015). SAM is synthesized by S-adenosylmethionine synthetase (SAMS) from Met. Under Fe deficiency, proteomic studies have shown that SAMSs are remarkably increased in abundance at the protein level among plant species. By 2DE-based proteomic study, Li et al., for the first time, reported the increase of a SAMS in abundance in tomato roots upon Fe deficiency (Li et al., 2008). Meanwhile, they found three Met synthases, which are associated with Met metabolism, were induced upon Fe starvation (Li et al., 2008). Subsequently, the abundance of SAMS was shown to increase in cucumber roots upon Fe starvation by means of 2DE approach (Li and Schmidt, 2010). Similarly, another proteomic study in cucumber roots showed two SAMSs, the *Populus trichocarpa* ortholog MAT1-a and the *Medicago sativa* ortholog, are up-regulated under Fe shortage (Donnini et al., 2010). While this study also reported another SAMS, the *Populus trichocarpa* ortholog MAT1-b, which was deceased in protein abundance upon Fe deficiency, and it could be the first case that SAMS was down-regulated upon Fe deficiency (Donnini et al., 2010). The down-regulation of SAMS might be due to the treatment and sampling time since Fe deficiency responses are rhythmic (Chen et al., 2013; Hong et al., 2013; Salome et al., 2013). Recently, for the second time, an ortholog of Arabidopsis SAMS2 was identified to be down-regulated from *Brassica napus* phloem sap upon Fe deficiency by using of 2DE technique; by means of Fe-affinity chromatography coupled with 1DE, the authors revealed two ACC oxidases (ACOs) that were induced in *Brassica napus* phloem sap upon Fe deficiency, which is consistent with the decrease of ACC content (Gutierrez-Carbonell et al., 2015). A putative SAMS, ortholog of AT1G78240, was reported to be up-regulated in *Medicago truncatula* under Fe limitation (Rodriguez-Celma et al., 2011). By combining HPLC-MS and iTRAQ, Lan et al. identified 4454 proteins in Arabidopsis roots and 2882 proteins were reliably quantified; of which, a suit of 101 proteins were identified as differentially accumulated in abundance upon Fe deficiency (Lan et al., 2011). Remarkably, this study revealed that six proteins associated with Met cycle, such as SAMS1, SAMS2, SAMS3, and SAMS4, the cobalmine-independent Met synthase ATMS1, and the S-adenosyl-L-homo-Cys hydrolase SAHH1, were found among the most abundant proteins, indicating the importance of this pathway in roots. Particularly, three out of four SAMSs showed an increase in protein abundance upon Fe deficiency, and two enzymes ARD2 (acireductone dioxygenase 2) and DEP1 (DEHYDRATASE-ENOLASE-PHOSPHATASE-COMPLEX 1), with a proposed function in the salvage of L-Met from methylthioadenosine (Figure 3), were also up-regulated by Fe shortage, underlying the importance of SAM synthesis and the sustainable Met cycle under Fe limiting conditions. Enhanced synthesis of SAM, combined with the up-regulation of NAS4, can lead to an increase of NA content, which is crucial for Fe homeostasis. Alternatively, increased SAM content would result in more ethylene production, which plays multiple roles in various biological processes by controlling the expression of downstream genes.

## Conclusions

Omic approaches have been widely employed to explore the responses of plant to Fe deficiency, and have uncovered diverse metabolic adaptations upon Fe starvation. A subset of conserved Fe-responsive genes and some common metabolic pathways have been revealed by transcriptome and proteome across a range of plant species. It has been clear that the concordance between the abundance of mRNA and their related proteins is not strong correlated (Lan et al., 2012a, 2013a; Li et al., 2013; Marmiroli et al., 2015; Dong et al., 2016). The integration of transcriptome and proteome is mandatory for generating a complete inventory of the components that are crucial for Fe homeostasis. Several core genes encoding proteins with unknown functions, which are robustly induced by Fe starvation and tightly co-expressed, require further validation. The involvement of ethylene in the morphological and physiological Fe deficiency responses in multiple plant species has been observed, and omic studies provide further molecular evidence that ethylene plays a role in the Fe deficiency responses of plants.

## Author contributions

All authors listed, have made substantial, direct and intellectual contribution to the work, and approved it for publication.

### Conflict of interest statement

The authors declare that the research was conducted in the absence of any commercial or financial relationships that could be construed as a potential conflict of interest.
