# Supplementary material for: The Understanding of the Plant Iron Deficiency Responses in Strategy I Plants and the Role of Ethylene in This Process by Omic Approaches
Source: Front Plant Sci. 2017 Jan 24;8:40. doi: 10.3389/fpls.2017.00040 (PMC5259694; doi:10.3389/fpls.2017.00040)
Supplement: Supplementary file 1 [file Table1.DOC]

Table S1 List is proteins associated with Methionine salvage cycle and ethylene synthesis in multiple species.

| **Proteins** | **Species** | | | | |
| --- | --- | --- | --- | --- | --- |
|  | *At* | *Mt* | *Sl* | *Bn* | *Cs* |
| **SAMS1** | Up, Lan et al. 2011;Pan et al. 2015 |  |  |  | Spot 1321 up, sopt 1341 down, Donnini et al. 2010 |
| **SAMS2** | Up, Lan et al. 2011 |  |  | Down, Gutierrez-Carbonell et al. 2015 | Up, Li et al 2010 |
| **SAMS3** | Up, Lan et al. 2011 |  |  |  |  |
| **Putative S-adenosylmethionine-**  **dependent methyltransferases** |  | Up, Ortholog of AT1G78240,Rodríguez-Celma et al. 2011 |  |  |  |
| **S-adenosyl methionine**  **synthase-like** |  |  | Up, Li et al. 2008 |  | Spot 1340 up, Donnini et al. 2010 |
| **ACO(Q09052),ACO3(Q08507)** |  |  |  | Up, Gutierrez-Carbonell et al. 2015 |  |
| **ARD2** | Up, Lan et al. 2011 |  |  |  |  |
| **DEP1** | Up, Lan et al. 2011 |  |  |  |  |

*At, Arabidopsis thaliana; Mt, Medicago truncatula ;Bn,Brassica napus;Cs, Cucumis sativus L; Sl, Solanum lycopersicum L*
